# Transmission models of *Mycobacterium ulcerans*: A systematic review

**DOI:** 10.1371/journal.pntd.0013376

**Published:** 2025-08-19

**Authors:** Rebecca Rasmussen, Katherine B. Gibney, Timothy P. Stinear, Jennifer A. Flegg, Patricia T. Campbell

**Affiliations:** 1 School of Mathematics and Statistics, The University of Melbourne, Melbourne, Victoria, Australia; 2 Department of Infectious Diseases, The University of Melbourne, at the Peter Doherty Institute for Infection and Immunity, Melbourne, Victoria, Australia; 3 Victorian Infectious Diseases Service, Royal Melbourne Hospital, at The Peter Doherty Institute for Infection and Immunity, Melbourne, Victoria, Australia; 4 Department of Microbiology and Immunology, The University of Melbourne at The Peter Doherty Institute for Infection and Immunity, Melbourne, Victoria, Australia; The University of Hong Kong, CHINA

## Abstract

**Background:** Buruli ulcer, caused by *Mycobacterium ulcerans*, is a neglected tropical disease affecting 33 countries worldwide. Elucidating the full transmission pathways of this infection remains an important active field of investigation, especially in Central and West Africa and Southeast Australia in the state of Victoria where disease burden is high. This systematic review (pre-registered on PROSPERO: CRD42023452944) provides an overview of mathematical transmission models of *M. ulcerans* and highlights future areas of investigation crucial to understanding transmission of Buruli ulcer and quantifying the impact of potential preventative interventions. **Methodology/principal findings:** We searched Scopus, PubMed, Embase and CAB abstracts on January $31^{\text{st}}$ 2025, and included studies which reported novel mechanistic models of *M. ulcerans* transmission. We qualitatively compared mathematical model structures, parameterisation methods, model analyses and author conclusions, and conducted a quality assessment of the studies using a modified Philips checklist. Twenty studies met the inclusion criteria; 18 performed theoretical analyses, while only five validated their model against empirical data, limiting conclusions and the implications for disease management. Seventeen studies focused on a water bug/fish/human system proposed for Central and West Africa with a diverse ranges of model structures. Three models described the mosquito/possum system of Southeast Australia using similar models, with none considering human populations. **Conclusions/significance:** This review highlights a large gap in modelling Buruli ulcer in Victoria: there are currently no models of this system that have been specifically formulated with data. Such models could be valuable for exploring and testing likely transmission scenarios. Future research should focus on developing models that incorporate local data and consider all potential transmission pathways to better understand the disease dynamics and evaluate potential interventions.

## Author summary

Buruli ulcer is a neglected tropical disease caused by the bacterium *Mycobacterium ulcerans*. It poses a significant public health challenge in both Central and West Africa and Southeast Australia—Buruli ulcer causes stigmatising disfigurements and disabilities which often lead to personal economic instability. Despite this, the transmission mechanisms of *M. ulcerans* to humans remain poorly understood, impeding the development of effective control and prevention strategies. In Australia, researchers identified that mosquitoes and possums play a role in *M. ulcerans* transmission to humans, however, the complete transmission cycle is not understood. In Central/West Africa, the transmission vector remains unconfirmed. Given this knowledge gap, mathematical models have been developed with the aim of clarifying infection mechanisms. Our study presents the first systematic review of such models. We analysed various model structures and their relevance to observed transmission patterns and found crucial gaps—there were no mosquito models for Africa and no human models for Australia. Additionally, we found that most studies did not validate their model with real-world data. Our review of *M. ulcerans* transmission models highlights important avenues for future research, both in the context of Buruli ulcer and for other diseases with complex environmental interactions or multiple transmission routes.

## Introduction

Buruli ulcer is a neglected tropical disease caused by the bacterium *Mycobacterium ulcerans*, involving the destruction of the skin and soft tissue. The disease has been documented in 33 countries worldwide [1], particularly in Central and West Africa [2, 3], and Southeast Australia [4]. Buruli ulcer presents a substantial public health burden: prevalence estimates in Central and West Africa range from 3.2 cases per 10,000 population in Côte d’Ivoire to 26.9 cases per 10,000 population in Benin [5]. Additionally, we are seeing a concerning geographic spread of Buruli ulcer in Southeast Australia [6]. Despite this, the mechanisms of transmission to humans have not been completely established [7], leading the WHO Buruli ulcer fact sheet to state “the mode of transmission is not known and there is no prevention for the disease” [1].

In Victoria, Australia, epidemiological investigations have identified the mosquito *Aedes notoscriptus* as a vector of *M. ulcerans* [8], and the common ringtail possum *Pseudocheirus peregrinus* as a mammal reservoir of the pathogen, shedding the bacteria into the environment through its faeces [8–10]. In contrast, the transmission dynamics in Central and West Africa remain less clear. While *M. ulcerans* has been detected in many aquatic taxa, including water bugs (*Belostomatidae* and *Naucoridae*) and mosquitoes [11–13], their role in the Central and West African transmission cycle is yet to be determined [7]. There are similarities in Buruli ulcer epidemiology across Africa and Australia: observed seasonal occurence of disease [14]; spatial overlap of Buruli ulcer cases in humans and environmental *M. ulcerans* [15]; and living near aquatic environments is associated with an increased risk of Buruli ulcer [16]. Importantly, experimental evidence has shown that subcutaneous inoculation with *M. ulcerans* is required for Buruli ulcer to develop in a mammalian host, not just contact with the skin (broken or unbroken) [17, 18]. This requirement, coupled with the close genetic links between the strains of *M. ulcerans* in Australia and Africa [19] and the fact that the bacterium is a specialist not a generalist [20], suggests that while the mammalian host may differ, the transmission mechanisms across both continents may be similar.

Despite these advances, important knowledge gaps remain in both geographical settings. In Southeast Australia, the complete transmission cycle, including the role of other potential vectors or reservoirs, is not fully understood. In African settings, the identity of the transmission vector(s) and potential animal reservoirs remain unconfirmed. Mathematical models are useful tools to explore these and other aspects of infection transmission. Such models are commonly used to clarify transmission dynamics, predict outbreaks, identify effective controls, and inform research priorities. For pathogens like *M. ulcerans*, mathematical models can help to disentangle the interactions between multiple biotic and abiotic factors that cannot be elucidated from observational data alone.

In this paper, we present the first systematic review of mathematical models of *M. ulcerans* transmission. The aims of this review are to qualitatively compare the mathematical models that have been used to describe the transmission dynamics of *M. ulcerans*, and to highlight any unanswered epidemiological questions that could be addressed by modelling. Principally, we aim to identify the relevance of existing *M. ulcerans* transmission model structures to observed transmission dynamics in Victoria, Australia.

## Materials and methods

We report on a systematic review of transmission models for *M. ulcerans* according to the Preferred Reporting Items for Systematic reviews and Meta-Analyses (PRISMA) statement [21]. The review was pre-registered on PROSPERO (ID number CRD42023452944) and can be accessed at www.crd.york.ac.uk/PROSPERO/display_record.asp?ID=CRD42023452944. A protocol for this systematic review was not published.

### Data sources and search strategy

We searched Scopus, PubMed, Embase and CAB Abstracts on August 21st 2023, with no language or publication date restrictions. The search was conducted again on January 31st 2025 and new studies were included before final analysis. In addition, we conducted forwards and backwards searches (on August 21st 2023 and again on January 31st 2025) using the identified articles and found papers outside of the chosen databases.

Our search consisted of terms to capture the pathogen, disease and model formulation. The same search strategy was used in each of the four databases (modified in structure to work with each database, see S1 Appendix). For example, in Scopus the following string was used:

TITLE-ABS-KEY (buruli OR mycobacterium OR bairnsdale OR mossman OR searls’) AND (ulcer*) AND (model* OR paramet* OR simulat* OR program* OR comput*).

### Eligibility criteria

Peer-reviewed studies were eligible for inclusion if they described a mathematical model of the *M. ulcerans* transmission mechanism—including mechanistic, compartmental, agent-based, spatial, or numerical models, both deterministic and stochastic. We did not include laboratory models, species distribution models or statistical models purely based on data. Unpublished manuscripts and conference abstracts were not eligible for inclusion.

### Screening and data collection

Screening and selection were undertaken in Covidence [22]. Two reviewers (RR and PC) screened the references independently, and resolved conflicts through discussion with JF. The two reviewers were blinded to each other’s decisions.

One reviewer (RR) extracted data from each included study, specifically: primary author, year of publication and the modelled location as well as model purpose, structure and parameterisation, and the results and conclusions of each study. These results included model outputs related to the basic reproduction number (*R*_0_), key parameters in sensitivity analyses and outcomes of any analyses. We included results of numerical simulations where they promoted conclusions unrelated to the information already extracted. For example, we excluded discussion of any numerical simulations related to *R*_0_, where *R*_0_ was an analytical result elsewhere in the paper.

### Data synthesis

In this review, we qualitatively compared mathematical model structures, parameterisation methods, model analyses and conclusions drawn by authors. We also assessed the applicability of the model choice in the context of present-day knowledge and the relevance of the model choice to observed transmission dynamics in Victoria, Australia. We grouped studies first by type of analysis, either being purely theoretical or containing some application to real-world data to compare high level model structures and analyses. Then we grouped the studies by the modelled location, either Central and West Africa or Southeast Australia for comparison of location-specific model features.

One reviewer (RR) used a modified Philips checklist [23] (excluding unrelated questions) for assessing the risk of bias in the included studies as well as the extent to which they answered their research question (see S2 Appendix), as recommended by the Cochrane Handbook [24].

### Infectious disease model terminology

For readers less familiar with mathematical models, we provide the following brief explanation of compartmental mechanistic models of infectious diseases to guide interpretation of the results of our review. These kinds of models make up the majority of the studies included in this review, and those that employ different model structures use similar concepts. For more in depth reading, see [25, 26].

*Compartments*: Compartmental models consist of populations broken down into mutually exclusive epidemiological stages (“compartments”). Relevant compartments may include:

- susceptible compartments (*S*), containing individuals of a species that have not yet been exposed to infection but are susceptible to infection
- exposed compartments (*E*) containing individuals who have been infected with a pathogen but are not yet capable of transmitting the infection
- infectious compartments (*I*) containing those capable of transmitting the infection
- recovered/removed compartments (*R*) containing those that are no longer capable of transmitting infection

Models may include other mutually exclusive compartments, for example a treatment compartment (*T*) containing individuals who are receiving treatment for the disease. Individuals from the populations in each compartment move through these stages at rates governed by a set of parameters.

For diseases that are spread via vectors such as mosquitoes, similar compartments to those above may be included for the vector population. Environmental sources of infection can also be modelled, typically as a separate compartment with which the host and vector populations come into contact. Susceptible individuals can become infected through contact with an infected organism or contaminated environment at rates that are either dependent on the density of the infected organism (density-dependent transmission) or independent of the organism density (frequency-dependent transmission).

*Reproduction number*: The basic reproduction number *R*_0_ is defined as the average number of new infections caused by a single infectious individual in a totally susceptible population and is specific to the pathogen and population [25]. If *R*_0_>1 then an outbreak of the infection will occur (on average) and if *R*_0_<1 then, on average, the infection will die out without an outbreak. Since *R*_0_ is typically straight-forward to calculate given a transmission model (at least numerically), it is often used as a proxy for the potential impact of the disease.

*Intervention analyses*: Interventions aimed at controlling either the spread of the pathogen or mitigating the severity of disease, given infection, are often incorporated in compartmental models. This may be achieved by changing parameter values governing flows between model compartments. For example “optimal control analyses”, where control variables are introduced into the model to represent means of mitigating the spread of the pathogen, can be used to optimise the effect of interventions. Alternatively, modellers may include additional compartments to allow different outcomes of infection. For example, inclusion of a treatment compartment as above may be used to allow for more rapid recovery of treated individuals. These techniques can inform potential avenues for real-world disease control.

The role of vectors in the transmission of an infectious disease may be either mechanical (carried between hosts on the body of the vector e.g. *Yersinia pestis* and fleas) or biological (vector becomes infected and must become infectious before transmission to a new host can occur e.g. *Plasmodium spp.* and mosquitoes). In the case of biological transmission, the life cycle of the vector and the natural history of the pathogen are modelled to capture the impact that their interaction has on transmission.

## Results

### Summary of studies included in the review

**Fig 1:**
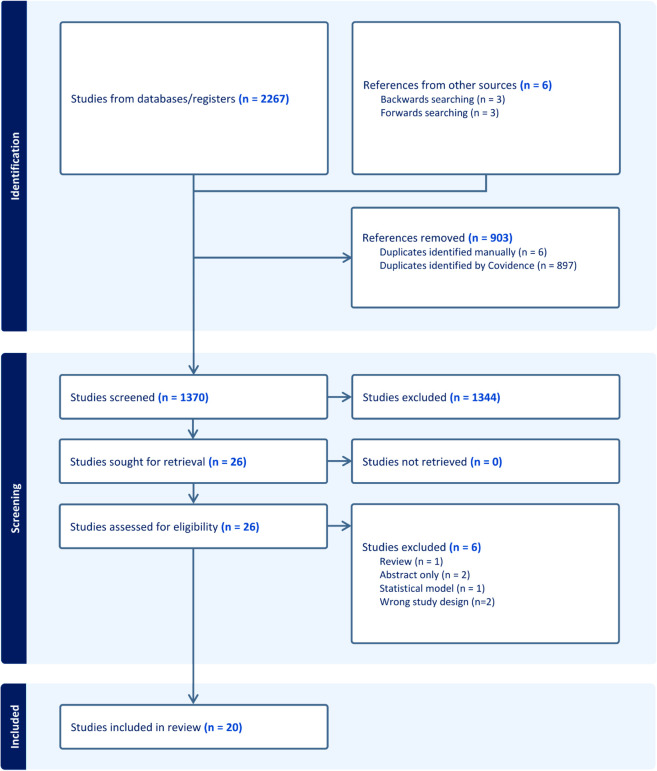
PRISMA flow chart. PRISMA flow chart depicting article selection process. Constructed with Covidence [22].

We performed a full text screening of 26 articles for eligibility for inclusion. Six of these were excluded because they were reviews [27], were abstract only [28, 29], detailed a generic transmission model instead of one specific to *M. ulcerans* [30, 31] or contained only a statistical model [32]. Of the 20 studies included in the systematic review, 14 were found in the database search, and a forwards and backwards search of these yielded a further six eligible studies (Fig 1). The following sections detail characteristics of the included models and results of the studies, for more summary tables and a quality assessment, see S2 Appendix.

### Characteristics of included models

**Fig 2:**
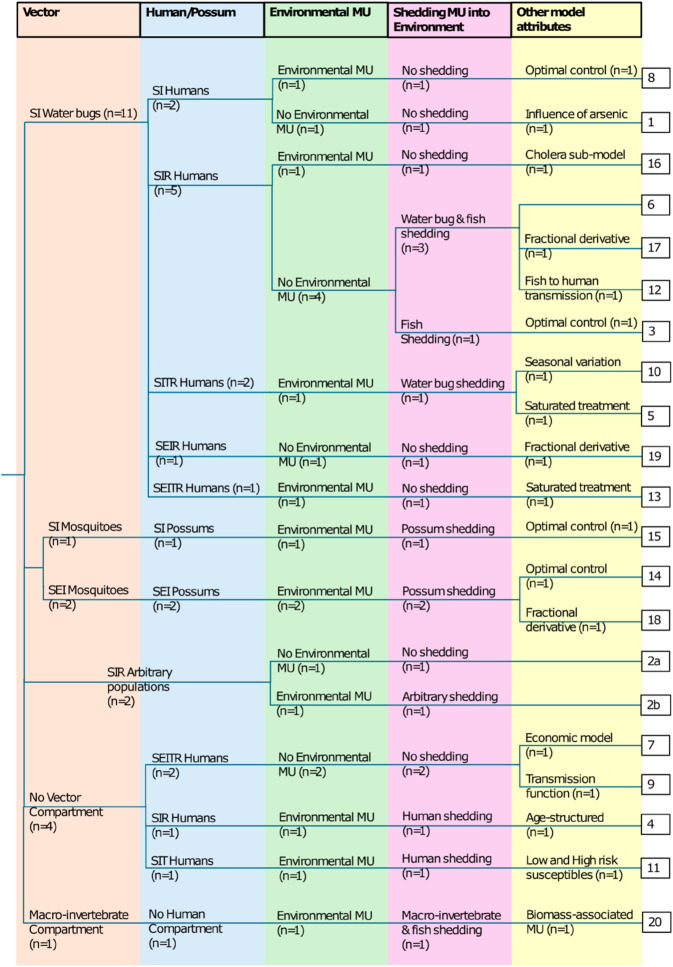
“Phylogenetic tree” of models. A “phylogenetic tree” representing the commonalities between models from the selected articles. Each “tree node” represents a differentiation in model structure. Model attributes, corresponding with the column headings, are found over each “branch”. The IDs for the models (listed in Table 1) described by each set of attributes are at the terminal end of each “branch”. Here we denote *M. ulcerans* as “MU” and describe model compartments with letter signifiers (for example, “SIR Humans” for a susceptible-infectious-recovered compartmental model for humans).

**Fig 3:**
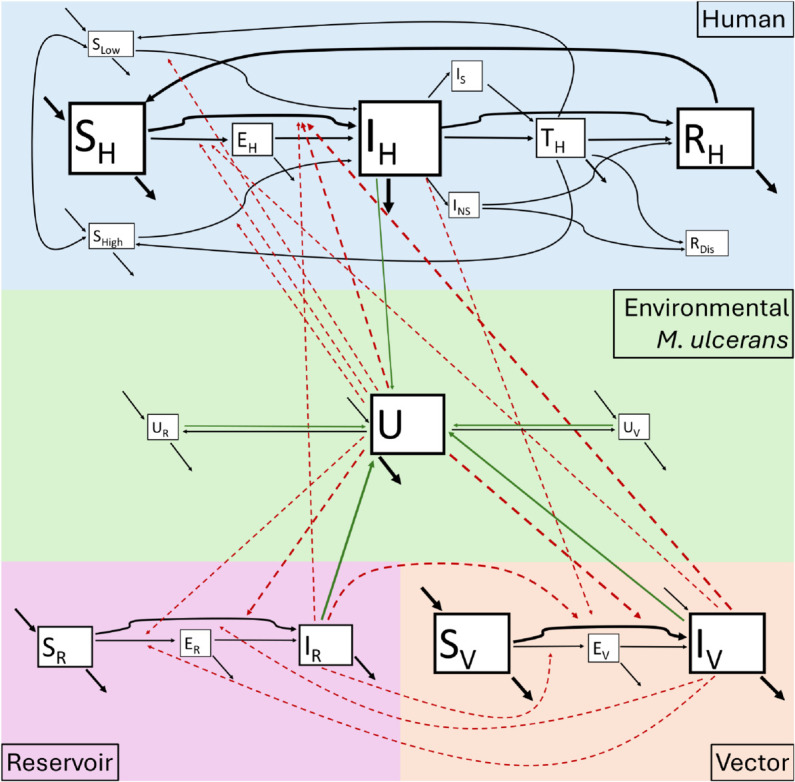
Compilation of model compartments. Compartment model diagram depicting the frequency of compartments in reviewed models and transition/transmission pathways. Compartments are susceptible (*S*), exposed (*E*), infectious (*I*), treated (*T*) and removed (*R*) with subscripts *H* for humans, *R* for reservoirs, *V* for vectors and compartment *U* for environmental *M. ulcerans*. *S*_*Low*_ and *S*_*High*_ represent susceptible individuals at low and high risk, respectively. *I*_*S*_ and *I*_*NS*_ represent infectious individuals seeking treatment or not seeking treatment. *R*_*Dis*_ represents individuals who acquire a disability as a result of having Buruli ulcer. *U*_*R*_ and $U_{V}$ represent reservoir and vector associated bacteria. Compartment box sizes represent the relative frequency of these compartments in the reviewed models. Black arrows indicate transitions of individuals between compartments, red dashed arrows represent *M. ulcerans* transmission routes and green arrows represent shedding of *M. ulcerans* from infectious compartments into the environment. Thickness of the arrows represent the relative frequency of pathways in the reviewed models. To see the IDs for the models (listed in Table 1) containing each pathway and compartment, see S3 Appendix.

The following description refers to model identification numbers (ID) assigned in Table 1, which provides an overview of the included *M. ulcerans* transmission models. Further, a “phylogenetic tree” demonstrating the commonalities between these models is provided in Fig 2 and a network of compartments used in the transmission models is provided in Fig 3.

**Table 1:** Overview of Buruli ulcer transmission models. The first column contains the article ID assigned for ease of reference and is used throughout this review—this is different from the article reference number shown in the second column. ID 2a refers to the base model in article 2, and 2b refers to the model presented in the supplimentary material of paper ID 2. Model characteristics present in model 2b and not in 2a are shown in brackets.

ID	Primary author, year, [reference]	Modelled location	Study aim	Model		
				Deterministic/ Stochastic	Populations	Forces of infection
1	Aidoo 2007 [33]	Ghana	Used a mathematical model to investigate the epidemiology of Buruli ulcer.	Deterministic	Humaneak Water bug	Water bug → Humaneak Human → Water bug
2a (2b)	Roche 2013 [11]	Ghana	Tested whether removal of keystone species in a multi-host system can control spread of disease.	Deterministic	Arbitrary specieseak (Environmental MU)	Arbitrary
3	Bonyah 2014 [34]	Central and West Africa	Analysed a *M. ulcerans* transmission model and applied optimal control theory to find the best way to prevent the spread of *M. ulcerans*, given costs.	Deterministic	Humaneak Water bugeak Fisheak Environmental MU	Water bug → Humaneak Fish → Water bugeak Environmental MU → Water bugeak Environmental MU → Fish
4	Bonyah 2014 [35]	Ghana	Developed an age-structured *M. ulcerans* model and provided some theoretical and numerical analysis of the model.	Deterministic	Humanseak Environmental MU	Environmental MU → Human
5	Bonyah 2014 [36]	Ghana	Theoretically modelled the challenges associated with the treatment/management of Buruli ulcer and the possible impacts thereof.	Deterministic	Humanseak Water bugseak Environmental MU	Water bug → Humaneak Environmental MU → Humaneak Environmental MU → Water bug
6	Nyabadza 2015 [37]	Ghana	Studied the dynamics of *M. ulcerans* in the presence of a preventive control strategy, while emphasizing the role of the vector (water bugs) and fish and their interaction with the environment.	Deterministic	Humaneak Water bugeak Fisheak Environmental MU	Water bug → Humaneak Fish → Water bugeak Environmental MU → Water bugeak Water bug → Fisheak Environmental MU → Fish
7	Garchitorena 2015 [38]	Districts ofeak Bankim andeak Akonolingaeak in Cameroon	Contributed to the economic analysis of rare-but-devastating diseases through specific models that account for feedback between economics and disease from individuals to the populations that they comprise.	Stochastic	Human	Arbitrary
8	Kimaro 2015 [39]	Ghana	Applied optimal control theory to the transmission dynamics of *M. ulcerans* infection.	Deterministic	Humaneak Water bugeak Environmental MU	Water bug → Humaneak Human → Water bugeak Environmental MU → Water bug
9	Garchitorena 2015 [40]	16 aquatic ecosystems across Bankim and Akonolinga	Assessed the relative contribution of two potential transmission routes—environmental and water bug transmission—to the dynamics of Buruli ulcer over space and time in two endemic regions of Cameroon.	Deterministic	Humans	Vector → Humaneak Environmental MU → Human
10	Assan 2017 [41]	Ghana	Incorporated seasonal factors into a mathematical model of *M. ulcerans* transmission to gain a deeper understanding of the short– and long– term evolution of *M. ulcerans*.	Deterministic	Humaneak Water bugeak Environmental MU	Water bug → Humaneak Environmental MU → Humaneak Environmental MU → Water bug
11	Edholm 2019 [42]	Ghana	Illustrated the case of pathogens that have only one transmission route—indirect through pathogens in the environment—using data and simulations for Buruli ulcer disease.	Deterministic	Humaneak Environmental MU	Environmental MU → Human
12	Nyarko 2020 [43]	Offin River in the Central Region of Ghana	Developed a transmission model of Buruli ulcer and helped to identify control measures that will minimize the disease in the study area.	Deterministic	Humaneak Water bug/Mosquitoeak Fish/Frogeak Environmental MU	Water bug/Mosquito → Humaneak Fish/Frog → Humaneak Fish/Frog → Water bug/Mosquitoeak Environmental MU → Water bug/eak Mosquitoeak Environmental MU → Fish/Frog
13	Momoh 2021 [44]	Central and West Africa	Explored the dynamics of Buruli ulcer with a mathematical model and optimal control intervention strategies.	Deterministic	Humaneak Water bugeak Environmental MU	Water bug → Humaneak Human → Water bugeak Environmental MU → Water bug
14	Chu 2021 [45]	Australia	Mathematically modelled *M. ulcerans* infections in possums for the purpose of exploring transmission and possible controls.	Deterministic	Possumeak Mosquitoeak Environmental MU	Mosquito → Possumeak Environmental MU → Possumeak Possum → Mosquito
15	Khan 2021 [46]	Australia	Created a mathematical model to understand the dynamics of *M. ulcerans* in possums.	Deterministic	Possumeak Mosquitoeak Environmental MU	Mosquito → Possumeak Environmental MU → Possumeak Possum → Mosquito
16	Zhao 2021 [47]	Amansie West in the Ashanti Region of Ghana	Used a mathematical model to explore the best strategy for controlling both *M. ulcerans* and cholera.	Deterministic	Humaneak Environmental Choleraeak Water bug	Water bug → Humaneak Environmental Cholera → Humaneak Human → Water bug
17	Ahmad 2022 [48]	Ghana	Aimed to find the environmental cause of the spread of Buruli Ulcer	Deterministic	Humaneak Fisheak Environmental MU	Water bug → Humaneak Fish → Water bugeak Environmental MU → Water bugeak Water bug → Fisheak Environmental MU → Fish
18	Farhan 2023 [49]	Australia	Mathematically modelled *M. ulcerans* infections in possums for the purpose of exploring transmission and possible controls.	Deterministic	Possumeak Mosquitoeak Environmental MU	Mosquito → Possumeak Environmental MU → Possumeak Possum → Mosquito
19	Fandio 2023 [50]	Cameroon	Formulated a new *M. ulcerans* compartmental model including latent period, standard force of infection, with both classical and fractional derivatives in the Caputo sense.	Deterministic	Humaneak Water bug	Water bug → Humaneak Human → Water bug
20	Sylla 2024 [51]	Not specified	Developed a metapopulation model to simulate pathogen transmission to capture how environmental bacteria abundance depends on regional dynamics of upstream catchment areas.	Deterministic	Fisheak Macro– invertebrateseak Environmental MUeak Fish– associated MUeak Macro– invertebrateeak – associated MU	Environmental MU attaches to fish to make fish-associated MU and likewise for macro– invertebrates

Of the 20 included studies, 15 papers modelled the water bug/human/fish dynamic in Central and West Africa (IDs 1,3– 13,16,17,19), three modelled the possum/mosquito dynamic in Australia (IDs 14,15,18), one (ID 2) modelled an arbitrary number of unspecified populations in Central and West Africa and one (ID 20) modelled a general system of *M. ulcerans*, macroinvertebrates and fish (Fig 2 and Table 1).

Of the 16 models based in Central and West Africa most (*n* = 12) focused on water bugs as vectors (predominantly Belostomatidae and Naucoridae) (IDs 1,3,5,6,8,10,12,13,16,17,19), while four did not consider a population of vectors (IDs 4,7,9,11). None of the Central and West African models explored the potential of Buruli ulcer being a mosquito-borne disease, although paper ID 2 did sample mosquitoes, as part of the data for their network model, and found both mosquitoes and water bugs PCR positive for *M. ulcerans* (Fig 2 and Table 1). All three Australian models considered populations of possums, mosquitoes and environmental *M. ulcerans* (Fig 2 and Table 1). Only two models that included vectors modelled an exposed period for them (only IDs 14,18), so most models have assumed that *M. ulcerans* is transmitted mechanically rather than biologically (which would require an extrinsic latency period, represented in models as a vector exposed period) (Fig 3).

Only four of the 15 models that included humans (IDs 1,3– 13,16,17,19) modelled an exposed period for them (IDs 7,9,13,19; Fig 3). In models where humans did not contribute to transmission, the absence of an exposed compartment meant the modelled human dynamics occurred 4–5 months earlier than they would have with an exposed compartment (representing an intrinsic latency period). In models where humans did contribute to transmission, either directly or by shedding, the absence of an exposed compartment may have inhibited the ability to realistically describe dynamics. Human shedding was only present in the models where there were no other compartments able to shed (no vectors or reservoirs, IDs 4 and 11; Fig 2)—so while human shedding may not be biologically relevant it may have been used to create non-trivial model dynamics when there were no other infectious compartments. The majority of models (*n* = 19) were deterministic, while one model (ID 7) used stochastic delays for compartmental transitions (Table 1).

### Theoretical analysis

#### Force of infection (FOI)

Of the models reviewed, eight imposed a frequency dependent FOI from infectious to susceptible individuals for all transmission routes (IDs 1,3,5,6,10,12,13,17). Of these eight models, seven modelled environmental *M. ulcerans*. All but one of these used a linear force of infection from environmental *M. ulcerans* to vectors and reservoir hosts dependent on the carrying capacity of the environmenteak (IDs 3,5,6,10,12,17), while ID 13 used a frequency dependent FOI for environmental *M. ulcerans* to water bugs. Seven models used density dependent FOI for all populations (IDs 2a,8,9,14,15,16,18), including all the possum/mosquito models. ID 19 used a density dependent FOI for transmission from humans to vectors and a frequency dependent FOI for transmission from vectors to humans (as per the Ross-Macdonald model of malaria transmission [52, 53]). Models that included environmental *M. ulcerans* transmission to humans all used a Michaelis-Menten function for the FOI, where the transmission rate plateaus as the level of environmental *M. ulcerans* increases (IDs 2b,4,5,10,11). Paper ID 20 assumed *M. ulcerans* in the environment attaches to fish and aquatic macro-invertebrates (assumed mechanical transmission) at a rate described by a Michaelis-Menten function.

#### Sensitivity analyses and reproduction numbers

Where sensitivity analyses were conducted for models focusing on human/water bug/fish populations, a high level of infection was most positively correlated with: rate of water bug transmission to humans and human transmission to water bugs (IDs 8,13); water bug population size (ID 5); probability of seeking treatment (ID 7); and rate of water bug and environmental *M. ulcerans* transmission to fish (ID 6). The same sensitivity analyses showed a high level of infection was most negatively correlated with environmental *M. ulcerans* decay rate (IDs 5,6,11); water bug death rate (ID 5,8,13) and rate of human uptake into treatment (IDs 11,13). These dependencies were reflected in the reproduction numbers of human/water bug/fish models (IDs 1,3-13,16,17,19) (Table 2, Fig 3). Transmission from the environment to humans was only present in the reproduction number for two of the models (IDs 4,11) despite featuring in five separate models (IDs 4,5,10,11,16). The duration of the human exposed period was a factor in only one reproduction number (ID 19) despite human exposed compartments being included in three other models (IDs 7,9,13,19) (Table 2 and Fig 3). Of the 15 human/water bug/fish papers (IDs 1,3– 13,16,17,19), we were able to obtain *R*_0_ values for 7 (IDs 3, 5, 10 – 13, 19). Four of these papers predicted a Buruli ulcer outbreak in their studied system (i.e. *R*_0_>1; ID 5, 11, 12, 19; Table 2).

**Table 2:** Basic reproduction numbers, theoretical analysis techniques and results (excluding paper IDs 2 and 9, which did not produce theoretical results). Parameter and reproduction number values are reported where available. Note parameter units have not been standardised as information was often limited. Reproduction number dependence (positive (+) or negative (-)) and sensitivity analysis correlations are shown in brackets. Here we denote *M. ulcerans* as “MU”.

ID	Primary author, year and reference	Reproduction number $R_{0}$ dependence, parameter notation and values (where available)	$R_{0}$ formula and value (where available)	Numerical results
1	Aidoo 2007 [33]	*R*_0_ depended on: density *m*, biting rate *a* of water bugs (+) water bug → human transmission rate *b* (+) environmental MU → water bug transmission rate *a*_1_ (+) difference between water bug birth/death rate *μ* and level of arsenic-causedeak vector-importation *α* (-) human birth/death rate *r* (-)	$R_{0}=\frac{maa_{1}b}{(\mu -\alpha )r}$	A higher level of arsenic in the environment is positively correlated with the long-term prevalence of human infection. Plotted relationship between Buruli ulcer prevalence and reproduction number and population number in epidemiological compartments over time.
3	Bonyah 2014 [34]	*R*_0_ depended on: environmental MU → fish transmission rate $\beta_{F}=0.5$ (+) fish shedding rate σ=0.8 (+) fish death rate $\mu_{F}=0.004$(-) environmental MU carrying capacity *K* = 10000 and decay rate $\mu_{E}=0.65$ (-)	$R_{0}=\sqrt{\frac{\beta_{F}\sigma }{K\mu_{E}\mu_{F}}}$ = 0.12	All parameter components of *R*_0_ were estimated by the authors (no methods given). We subsequently used these to calculate *R*_0_. Optimal control analysis showed a combination of all controls optimally decreased infections.
4	Bonyah 2014 [35]	*R*_0_ depended on: environmental MU → human transmission rate $\beta_{H}=0.00065$ (+) human birth rate and survival probability Λ=0 (+) environmental MU decay rate $\delta_{v}=0.2$ (-) human death rate $\mu_{H}=0.45$ (-)	*R*_0_ = $\frac{\beta_{H}\Lambda }{\mu_{H}\delta_{V}}\int_{0}^{\infty }\eta_{1}(a)\pi (a)\;da$ = 0, where π(a)= $e^{-u(a)}e^{\int_{0}^{a}(\rho_{1}(\nu )-g(\nu ))\;d\nu }$	Parameter components of *R*_0_ were either estimated by authors (Λ and $\delta_{v}$, no methods given) or taken from [54] ($\beta_{H}$ and $\mu_{H}$). We subsequently used these to calculate *R*_0_. There was no disease outbreak under the parametrisation used.
5	Bonyah 2014 [36]	*R*_0_ depended on: water bug shedding rate α=0.006 and population size $N_{W}=1\times 10^{6}$ (+) environmental MU → water bug transmission rate $\beta_{3}=0.09$ (+) environmental MU carrying capacity $K_{d}=6.5\;\times \;10^{5}$ and decayeak rate $\mu_{d}=0.02$ (-) water bug death rate $\mu_{W}=0.025$ (-)	$R_{0}=\frac{\alpha N_{W}\beta_{3}}{K_{d}\mu_{d}\mu_{W}}=1.6492$	Several parameter sets were used in the numerical simulations, we have reported those used to calculate *R*_0_. Parameter components of *R*_0_ were either obtained from [33] ($\mu_{W}$) or assumed by the authors (methods not given). Sensitivity analysis showed reproduction number was most strongly correlated with: water bug shedding rate (0.9) environmental MU → water bug transmission rate (0.2) environmental MU decay rate (-0.25) water bug death rate (-0.2)
6	Nyabadza 2015 [37]	*R*_0_ depended on: fish → water bug transmission rate $\beta_{V}=6.5\times 10^{-5}$ (+) environmental MU → water bug transmission rate $\eta_{V}\beta_{V}=9.75\times 10^{-5}$ (+) water bug → fish transmission rate $\beta_{F}=5\times 10^{-5}$ (+) environmental MU → fish transmission rate $\eta_{F}\beta_{F}=3\times 10^{-5}$ (+) water bug $N_{V}$ and fish $N_{F}=10N_{V}$ population sizes (+) fish $\sigma_{F}=0.0075$ and water bug $\sigma_{V}=0.0009$ shedding rate (+) fish death rate $\mu_{F}=0.0075$ (-) water bug death rate $\mu_{V}=0.15$ (-) environmental MU decay rate $\mu_{E}=0.06$ and carrying capacity *K* (-)	$R_{0}=\frac{N_{F}\eta_{F}\sigma_{F}\beta_{F}}{K\mu_{E}\mu_{F}\mu_{V}}+\frac{N_{V}\eta_{V}\sigma_{V}\beta_{V}}{K\mu_{E}\mu_{V}^{2}}+\frac{\beta_{F}\beta_{V}}{K\mu_{E}\mu_{F}\mu_{V}^{2}}(K\mu_{E}\mu_{V}+N_{F}\sigma_{F}\eta_{V}+N_{V}\sigma_{V}\eta_{F})$	All parameter components of *R*_0_ were estimated by authors (no methods given). There was not enough information provided to calculate *R*_0_. Sensitivity analysis showed environmental MU concentration was most strongly correlated with: water bug → fish transmission rate (0.78) environmental MU → fish transmission rate (0.67) ratio of number of water bugs to fish (0.52) environmental MU decay rate (-0.73) fish death rate (-0.70)
7	Garchitorena 2015 [38]	None	None	Numerical simulations with different values for treatment effectiveness and time to seek treatment. The poorest subgroups of the population were impacted more economically by Buruli ulcer-caused disability, which increased economic inequality. Improving treatment (preventing more disabilities) increased wealth equality. Decreasing the time to seek treatment further increased wealth equality. Sensitivity analysis showed mean wealth was most strongly correlated with: probability of seeking treatment (0.73) human birth/death rate (0.33) reduction of human capital by disability (-0.37) minimum transmission rate (-0.32) probability of disability with treatment (-0.12)
8	Kimaro 2015 [39]	*R*_0_ depends on: water bug → human transmission rate $\beta_{1}=0.0014$ (+) human → water bug transmission rate $\beta_{3}=0.002$ (+) human *N*_*H*_ and water bug *N*_*W*_ population size (+) human *r*_1_ = 0.03 and water bug *r*_2_ = 0.002 birth/death rate (-)	$R_{0}=\sqrt{\frac{\beta_{1}\beta_{3}N_{H}N_{W}}{r_{1}r_{2}}}$	All parameter components of *R*_0_ were estimated by authors (no methods given). There was not enough information provided to calculate *R*_0_. Sensitivity analysis showed reproduction number was most strongly correlated with: water bug → human transmission rate (0.50) human → water bug transmission rate (0.50) human birth/death rate (-0.50) water bug birth/death rate (-0.50) Optimal control analysis showed a combination of all controls optimally decreased infections.
10	Assan 2017 [41]	Calculated *R*_0_ and time-averaged reproduction number *[R*_0_*]*: *[R*_0_*]* depended on: average environmental MU → water bug transmission rate $\hat{\beta_{3}}=0.09$ (+) average water bug shedding rate $\hat{\alpha }=0.00615K_{m}/N_{B}$ (+) water bug population size *N*_*B*_ (+) water bug death rate $\mu_{B}=0.8$ (-) environmental MU carrying capacity *K*_*m*_ and decay rate $\mu_{m}=0.15$ (-) The time-averaged reproduction number underestimated the infection risk when compared to the basic reproduction number i.e. $[R_{0}]<R_{0}$	$[R_{0}]=\frac{{\hat{\beta }}_{3}\hat{\alpha }N_{B}}{K_{m}\mu_{b}\mu_{m}}$eak = 0.0046.eak No analytical formeak for *R*_0_ was given.	Parameter components of *R*_0_ were taken from [33, 36]. We subsequently used these to calculate *R*_0_. Plotted relationship between reproduction numbers and transmission rate from environmental MU to water bugs and population numbers in epidemiological compartments over time.
11	Edholm 2019 [42]	*R*_0_ depended on: environmental MU growth rate *r* = 0.001 (+) environmental MU → human transmission rate (higheak $\beta_{H}=1.47\times 10^{-5}$ and low risk $\beta_{L}=1.00\times 10^{-10}$) (+) human birth $\pi_{h}=2.74^{3}$ and shedding rate $\eta =3.37\times 10^{-6}$ (+) environmental MU decay rate δ=0.00563 (-) half-saturation constant for environmental MU *k* = 0.25 (-) human death rate $\mu =4.5\times 10^{-5}$ and rate of uptake into treatmenteak γ=0.01 (-)	$R_{0}=\frac{r}{\delta }+$ $\left(\beta_{L}(\alpha_{2}+\mu )+\beta_{H}\alpha_{1}\right)$eak $\frac{\eta \pi_{h}}{\mu \delta k(\gamma +\mu )(\alpha_{1}+\alpha_{2}+\mu )}$eak = 1.552	*R*_0_ was calculated and parameter components of *R*_0_ were estimated by authors through model fitting (minimising difference between simulations and data). Sensitivity analysis showed the total number of infected individuals was most highly correlated with: rate of movement from the low to high risk populationseak for susceptible humans (0.93) environmental MU → high risk humans transmissioneak rate (0.92) rate of movement from the high to low risk populationseak for susceptible humans (-0.94) environmental MU decay rate (-0.93) rate of uptake into treatment (-0.49) environmental MU half-saturation constant (-0.48)
12	Nyarko 2020 [43]	*R*_0_ depended on: fish → water bug transmission rates $\beta_{V}$ (+) environmental MU → water bug transmission rates $\eta \beta_{V}$ (+) water bug shedding rate $\sigma_{V}$ and population size $P_{V}$ (+) environmental MU carrying capacity *K* and decay rate $\mu_{E}$ (-) water bug birth/death rate $\mu_{V}$ (-)	$R_{0}=\frac{\eta \beta_{V}P_{V}\sigma_{v}}{K\mu_{V}\mu_{E}}$ = 1.208	*R*_0_ was calculated although the parameterisation was unclear and the formula used appeared inconsistent with the model. Endemic equilibrium was reached after about 200 days.
13	Momoh 2021 [44]	*R*_0_ depended on: water bug biting rate ρ=0.08 (+) water bug → human transmission rate $\beta_{1}=0.4$ (+) human → water bug transmission rate $\beta_{3}=0.0015$ (+) rate of uptake into treatment σ=0.4 (-) human $\mu_{H}=0.004566$ and water bug $\mu_{W}=0.06$ natural death rate (-) human disease-induced death rate δ=0.005 (-)	$R_{0}=\sqrt{\frac{\rho^{2}\epsilon \beta_{1}\beta_{3}}{\mu_{w}(\epsilon +\mu_{H})(\sigma +\mu_{H}+\delta )}}$ = 0.012	Parameter components of *R*_0_ were either assumed by the authors (*δ* and *ε*) or taken from [34, 36, 39] (the rest). We subsequently used these to calculate *R*_0_. Sensitivity analysis showed reproduction number was most strongly correlated with: water bug biting rate (1) water bug → human transmission probability (0.5) human → water bug transmission probability (0.5) water bug death rate (-0.5) rate of uptake into treatment (-0.39) Optimal control analysis showed a combination of all controls optimally decreased infections.
14	Chu 2021 [45]	*R*_0_ depended on: possum shedding ϵ=0.07 and birth rate $\Pi_{m}=100$ (+) mosquito birth rate $\Pi_{V}=1000$ (+) environmental MU → possum transmission rate $\eta_{3}=0.01$ (+) mosquito → possum transmission rate $\eta_{1}=0.2$ (+) possum → mosquito transmission rate $\eta_{2}=8\times 10^{-5}$ (+) possum $1/\psi_{m}=5$ and mosquito $1/\psi_{V}=500$ latency period (-) environmental MU decay rate π=0.07 (-) possum $\nu_{m}=4.57\times 10^{-5}$ and mosquito $\nu_{V}=0.07$ natural death rate (-) possum disease– induced death rate $\nu_{1}=0.07$ (-)	$R_{0}=\frac{\epsilon \eta_{3}\psi_{m}\Pi_{m}}{2\pi l_{1}l_{2}\nu_{m}}$eak $+\sqrt{\frac{\frac{\epsilon^{2}\eta_{3}^{2}\psi_{m}^{2}\Pi_{m}^{2}}{4\pi^{2}l_{1}^{2}l_{2}^{2}\nu_{m}^{2}}+}{\frac{\eta_{1}\psi_{m}\Pi_{m}\eta_{2}\psi_{V}\Pi_{V}}{l_{1}l_{2}l_{3}\nu_{m}\nu_{V}^{2}}}}$ = very large *R*_0_, where $l_{1}=\psi_{m}+\nu_{m}$, $l_{2}=\nu_{m}+\nu_{1}$, $l_{3}=\nu_{V}+\psi_{V}$.	Parameter components of *R*_0_ were either assumed by the authors ($\eta_{1}$, $\eta_{2}$, $\psi_{m}$, $\psi_{V}$, $\Pi_{m}$, $\Pi_{V}$) or taken from [34, 36, 37] (the rest). These parameter values and the *R*_0_ formula provided resulted in an *R*_0_ value out of the biologically plausible range. Optimal control analysis showed a combination of all controls optimally decreased infections.
15	Khan 2021 [46]	*R*_0_ depended on: possum shedding $\alpha_{E}=0.07$ and birth rate $\Lambda_{m}=2$ (+) mosquito birth rate $\Lambda_{V}=5$ (+) environmental MU → possum transmission rate $\alpha_{m}=0.01$ (+) mosquito → possum transmission rate $\beta_{m}=0.2$ (+) possum → mosquito transmission rate $\beta_{V}=0.09$ (+) environmental MU decay rate $\mu_{E}=0.07$ (-) possum disease-induced mortality rate $\delta_{m}=0.07$ (-) possum $\mu_{m}=4.57\times 10^{-5}$ and mosquito $\mu_{V}=0.07$ death rate (-)	$R_{0}=\sqrt{\frac{\frac{\Lambda_{m}\alpha_{E}\alpha_{m}}{\mu_{E}\mu_{m}(\delta_{m}+\mu_{m})}+}{\frac{\Lambda_{m}\beta_{m}\beta_{v}\Lambda_{v}}{\mu_{m}\mu_{v}^{2}(\delta_{m}+\mu_{m})}}}$ = 2.16	Parameter components of *R*_0_ were either assumed by the authors ($\Lambda_{m}$, $\Lambda_{v}$, $\beta_{m}$, $\beta_{v}$) or taken from [34, 36, 37] (the rest). We subsequently used these to calculate *R*_0_. Optimal control analysis showed a combination of all controls optimally decreased infections.
16	Zhao 2021 [47]	Calculated reproduction number for both the Buruli ulcer sub-model (*R*_0,*sub*_) and the Buruli ulcer-cholera co-infection model (*R*_0_): *R*_0,*sub*_ depended on: human $\pi_{h}=100$ and water bug $\pi_{V}=1000$ birth rate (+) water bug → human transmission rate $\beta_{h}=0.025$ (+) human → water bug transmission rate $\beta_{v}=0.005$ (+) human duration of infectiousness 1/η=20 (+) human disease-induced death rate κ=0.07 (-) human $\mu_{h}=4\times 10^{-5}$ and water bug $\mu_{v}$ death rate (-) *R*_0_ was functionally the same as *R*_0,*sub*_ except when the cholera sub-model reproduction number was larger—then *R*_0_ depended on some of the cholera parameters and positively on human birth, negatively on human death.	$R_{0,sub}=\sqrt{\frac{\pi_{h}\pi_{V}\beta_{h}\beta_{v}}{\mu_{h}\mu_{v}^{2}(\eta +\mu_{h}+\kappa )}}$	Parameter components of *R*_0_ were either assumed by the authors ($\beta_{v}$) or taken from [37, 55] (the rest). There was not enough information provided to calculate *R*_0_. Optimal control analysis showed a combination of all controls optimally decreased infections.
17	Ahmad 2022 [48]	*R*_0_ depended on: water bug $\sigma_{V}=0.63$ and fish $\sigma_{F}=0.63$ shedding rate (+) fish population size *N*_*F*_ (+) fish → water bug transmission rates $\beta_{V}=0.79$ (+) environmental MU → water bug transmission rates $\eta_{V}\beta_{V}=1.93$ (+) water bug → fish transmission rates $\beta_{F}=0.76$ (+) environmental MU → fish transmission rates $\eta_{F}\beta_{F}=1.86$ (+) fish death rate $\mu_{F}=2.79\times 10^{-4}$ (-) environmental MU carrying capacity *K* and decay rate $\mu_{E}$ (-)	$R_{0}=\frac{N_{F}\eta_{F}\sigma_{F}\beta_{F}}{K\mu_{E}\mu_{F}\mu_{V}}+\frac{N_{V}\eta_{V}\sigma_{V}\beta_{V}}{K\mu_{E}\mu_{V}^{2}}+\frac{\beta_{F}\beta_{V}}{K\mu_{E}\mu_{F}\mu_{V}^{2}}(K\mu_{E}\mu_{V}+N_{F}\sigma_{F}\eta_{V}+N_{V}\sigma_{V}\eta_{F})$	Parameter components of *R*_0_ were either taken from [34] ($\mu_{V}$) or assumed by authors (the rest). There was not enough information provided to calculate *R*_0_. A higher fractional derivative order resulted in faster time dynamics.
18	Farhan 2023 [49]	*R*_0_ depended on: possum shedding $\alpha_{E}=0.07$ and birth rate $\Lambda_{m}=100$ (+) mosquito birth rate $\Lambda_{V}=1000$ (+) environmental MU → possum transmission rate $\alpha_{m}=0.65$ (+) mosquito → possum transmission rate $\beta_{m}=0.7$ (+) possum → mosquito transmission rate $\beta_{V}=0.74$ (+) possum $1/\theta_{m}=50$ and mosquito $1/\delta_{V}=500$ latency period (-) environmental MU decay rate $\mu_{E}=0.07$ (-) possum $\mu_{m}=4.57\times 10^{-5}$ and mosquito $\mu_{V}=0.07$ death rate (-) possum disease– induced death rate $\delta_{m}=0.07$	$R_{0}=\frac{\alpha_{E}\alpha_{m}\theta_{m}\Lambda_{m}}{2k_{1}k_{2}\mu_{E}\mu_{m}}+\sqrt{\frac{(\frac{\alpha_{E}\alpha_{m}\theta_{m}\Lambda_{m}}{2k_{1}k_{2}\mu_{E}\mu_{m}}){\;}^{2}}{\frac{\beta_{m}\theta_{m}\Lambda_{m}\beta_{V}\delta_{V}\Lambda_{V}}{k_{1}k_{2}k_{3}\mu_{m}\mu_{V}^{2}}}}$ = very large *R*_0_, where $k_{1}=\theta_{m}+\mu_{m}$, $k_{2}=\mu_{m}+\delta_{m}$, $k_{3}=\mu_{V}+\delta_{V}$.	Parameter values for $\alpha_{m}$, $\beta_{m}$ and $\beta_{V}$ were varied (within the same order of magnitudes) by the authors for simulations. We have reported the values used that give the highest *R*_0_ value. Parameter components of *R*_0_ were assumed by authors (no methods given). These parameter values and the *R*_0_ formula provided resulted in *R*_0_ values out of the biologically plausible range. A higher fractional derivative order resulted in slower time dynamics.
19	Fandio 2023 [50]	*R*_0_ depended on: water bug birth rate $\pi_{V}=1.7\times 10^{5}$ (+) water bug → human transmission rate $\beta_{h}=0.78$ (+) human → water bug transmission rate $\beta_{V}=0.037$ (+) human human duration of infectiousness 1/η=1.31 (+) human latency period $1/\gamma_{1}=2.08$ (-) human birth rate $\pi_{h}=58.7$ and disease-induced death rate *K* = 0.2 (-) human $\mu_{h}=0.02$ and water bug $\mu_{V}=0.59$ death rate (-)	$R_{0}=\sqrt{\frac{\frac{\beta_{V}\beta_{h}\mu_{h}\pi_{V}}{\pi_{h}\mu_{V}}}{\frac{\gamma_{1}}{\left(\mu_{h}+\gamma_{1})(K+\mu_{h}+\eta \right)}}}$ = 2.0843	*R*_0_ was calculated and parameter components of *R*_0_ were estimated by authors through model fitting (least squares curve fitting).
20	Sylla 2024 [51]	None	None	Biomass MU productivity was more important to environmental MU levels than the ability of the biomass to disperse.

Among the possum/mosquito models, there were no sensitivity analyses conducted (IDs 14,15,18). Reproduction numbers most frequently positively depended on: the rate of possum shedding; possum and mosquito birth; and transmission from environment to possum mosquito to possum and possum to mosquito. These reproduction numbers most frequently negatively depended on the rate of possum and mosquito death (Table 2). It is important to note that there was limited variation within these possum/mosquito models—paper IDs 14 and 18 share the same reproduction number (same model construction) and ID 15 only differs in having no exposed period (Fig 3). The parameter values considered in paper IDs 14 and 18 resulted in biologically implausible values of *R*_0_ ($R_{0}>10^{3})$, whereas the *R*_0_ value calculated for paper ID 15 was biologically relevant, and predicted an outbreak (*R*_0_ = 2.16, Table 2).

#### Optimal control analysis

Across the board, when authors investigated optimal control variables in their system (IDs 3,4,8,13,14,15,16), where more controls were applied simultaneously, there was less disease. These analyses were not used in conjunction with data and so, despite the conclusion that we should implement as many controls as possible, this lacks support from real-world data or experiments.

### Applied models (IDs 2,6,9,11,19)

Here we report results and conclusions from studies where they have applied their models to a data set (Table 3). For further analysis of model applications by authors see S2 Appendix.

**Table 3:** Applied analysis techniques and results.

ID	Primary author, year and reference	Data source	Analysis	Results
2	Roche 2013 [11]	Data from Roche 2013 [11]; sampled 27 waterbodies, associated with Buruli ulcer in human communities in southern Ghana, for PCR positive invertebrates in June 2004 and August 2005.	Used model fitting (iterative estimation algorithm) to *M. ulcerans* prevalence in aquatic taxa to estimate the transmission rates between each taxon: iteratively compared model and data pathogen prevalence to estimate contact rates and network levels.	The most common taxa across the 27 different sites were not necessarily those that have strongest influence on *M. ulcerans* transmission. The authors used networks to show that removing one single taxon, the Oligochaeta worms, significantly decreased *M. ulcerans* prevalence in the models (by 15%–55%).
6	Nyabadza 2015 [37]	Ashanti Regional Disease Control Office; Buruli ulcer cases in Ghana per 100,000 people between the years 2003–2012 (Ghana statistical service).	Used model fitting (least squares curve fit) of Buruli ulcer yearly incidence to estimate eight parameters.	Model proved a reasonable fit to yearly Buruli ulcer incidence over 10 years. However, data seem to suggest an endemic equilibrium has been reached in the population and so the model fitting produced some unreasonable parameter values—fit predicted 5 times more humans than water bugs and 10 times more fish than water bugs.
9	Garchitorena 2015 [40]	Monthly Buruli ulcer incidence in Akonolinga (Camaroon) from 2002– 2012 [60], and water body and aquatic fauna sampling in Cameroon from June 2012 to May 2013 [13].	Used model fitting (maximising log-likelihood) to median monthly Buruli ulcer incidence to determine water bug and environmental *M. ulcerans* transmission rates to humans; Sensitivity analysis.	Results from the model fitting indicated that environmental transmission plays a larger role than, but does not completely rule out, water bug transmission of *M. ulcerans* to humans. Sensitivity analysis showed that if the median time to seek treatment of Buruli ulcer patients was higher than the 1– 4 month range (initially considered based on the data), then water bug transmission could play a role, contributing up to 20% of the infections at specific times of the year in the best model fit.
11	Edholm 2019 [42]	Monthly Buruli ulcer incidence in Ghana for 2008– 2015 from The Ministry of Health, Ghana health service.	Used model fitting (minimising difference between simulations and data) to monthly incidence to estimate 12 parameters.	The model fit quite well to data (especially if high and low risk susceptible populations are considered) and was consistent with a disease outbreak.
19	Fandio 2023 [50]	Yearly Buruli ulcer incidence in Cameroon for the period 2001–2014 [61].	Used model fitting (least squares curve fit) to yearly incidence for parameter estimation. Numerical simulation of fractional derivative model.	Model fit to data was adequate, but some parameter values were not biologically plausible: the predicted average life expectancy of someone living with Buruli ulcer was 5 years from infection (the disease is rarely fatal); the predicted latency period for *M. ulcerans* was 2 years (in reality closer to four months); and the predicted lifespan for the water bugs was around two years (closer to one year). The calculated strength number (*A*_0_ = 17) was greater than zero so the modelled number of infections can have multiple waves. Decreasing the fractional derivative seemed to decrease peak of humans exposed to *M. ulcerans* but increase long term numbers of human Buruli ulcer cases when fitting to data.

Paper IDs 6, 11 and 19 all fit their models to human incidence data in order to estimate several unknown model parameters. These models fit the data reasonably well, however all three model fits resulted in parameters seemingly outside the bounds of biological plausibility. For instance, paper ID 6 predicted that: the population of water bugs is a fifth as big as the human population; it takes an average of 17 days for humans to recover from Buruli ulcer (as opposed to months [1]); there are 10 times as many fish as water bugs; water bugs only live for seven days on average (six months to a year [56]); and fish only live for 20 days (at least eight weeks [57]). Paper ID 11 predicted that humans take an average of six days to recover from Buruli ulcer with treatment (several months [3]) and that the half-saturation constant is negligible compared to the initial environmental *M. ulcerans* concentration and so the transmission rate to humans is not modified by this concentration. Paper ID 19 predicted that the average life expectancy of someone living with Buruli ulcer is five years from infection (the disease is rarely fatal [1]), and the predicted latency period for *M. ulcerans* in humans is two years (closer to four months [58, 59]).

Paper IDs 2 and 9 both made biological conclusions based on their model outputs. Paper ID 9 used their model to predict that environmental transmission is responsible for more human Buruli ulcer cases than water bug transmission. Paper ID 2 used their model to investigate which invertebrate taxa are more crucial to the transmission cycle and found that Oligochaeta—a taxon of worms—could play an integral part in the transmission network. They predicted that the removal of Oligochaeta from the system would significantly decrease the *M. ulcerans* prevalence in the system. Both results contradict the widely assumed role of water bugs in transmission of *M. ulcerans* in Africa by other included studies.

Grouping the applied papers by the location modelled (Ghana: IDs 6 and 11, Cameroon: IDs 9 and 19) and comparing common parameters we see little similarity in values (see S4 Appendix). IDs 6 and 11 only share similar human death rates and these are taken from demographic data and not estimated through model fitting. IDs 9 and 19 do not have any similar parameter values. The lack of similarity in fit parameters indicates that model outputs are quite sensitive to the model structure.

## Discussion

This systematic review is the first to examine transmission model structures and assumptions of the dynamics of Buruli ulcer, a neglected tropical disease. We identified 20 studies that developed mechanistic *M. ulcerans* transmission models, focusing primarily on the dynamics in Central and West Africa (mostly Ghana and Cameroon) or Southeast Australia, despite reports of the disease in 33 countries [1]. Models of Central and West Africa predominantly explored aquatic transmission cycles, whereas Australian models focused on mosquito vectors and possum hosts. Most models were theoretical with few attempts to validate against empirical data, and those that did may not have fully captured the primary mechanisms of transmission (see S2 Appendix for quality assessment).

The reviewed studies included many variations on the multi-population compartment model and in general, these adhere to the known epidemiology of Buruli ulcer. The majority of models included a vector compartment, reflecting the evidence for transmission of *M. ulcerans* via biting vectors [17, 18] and most models treated humans as dead-end hosts. However, there were deviations from these established mechanisms where models prioritized non-trivial dynamics in simpler frameworks over real-world applicability. The majority of studies formulated a reproduction number for their model but only half of the reviewed studies provided enough information to calculate an *R*_0_ value. Considering that all papers were focused on systems with Buruli ulcer outbreaks, only four studies resulted in biologically plausible reproduction numbers; others either predicted no outbreak at all or were much larger than is biologically relevant. This may further indicate a limitation in the applicability of some of the reviewed frameworks.

Models for Central and West Africa focused on the interactions between water bugs, fish, and humans. These models reflected key aspects of African Buruli ulcer epidemiology, such as age-related differences in disease (ID 4) [62, 63] and seasonal peaks in incidence [13, 14] modeled through dynamic transmission and shedding rates (ID 10). Although these structural frameworks could be combined with data to test existing transmission hypotheses, relatively few authors utilized data for model validation. Given the largely unknown transmission mechanisms, these authors’ hypotheses remain untested. Furthermore, no explicit mosquito models were developed for the African context, despite ongoing debates about the routes of transmission, particularly regarding water bugs [2, 7]. The conclusions of article IDs 2 and 9 add to the body of evidence that vectors other than water bugs may warrant more consideration in Africa. With sufficient data, a mathematical model of appropriate structure could be used to further test these potential transmission routes.

In contrast, the Victorian context in Southeast Australia offers a more established understanding of local *M. ulcerans* transmission, which was reflected in the similarities between model structures. Existing models involve interactions among mosquitoes, possums, and the environment. Notably, IDs 14 and 18 incorporated an exposed compartment for possums, acknowledging the long latency period associated with the infection. While these models provide a foundation for exploring environmental dynamics, they do not explore transmission to humans and the resulting dynamics, and none were validated against empirical data. This highlights a significant gap in modelling *M. ulcerans* transmission in Victoria; specifically formulated models in combination with data are essential to address key transmission hypotheses. Current models for Africa and Australia involve different transmission cycles, but the strength of evidence supporting the involvement of hosts and vectors differs between the settings. The literature suggests an Australian model should capture a mosquito, vertebrate host and human cycle. Whether a similar transmission cycle exists in Africa could be explored using a modelling framework.

Additionally, no models have been created for *M. ulcerans* transmission in settings other than Victoria and Central and West Africa despite Buruli ulcer being documented in 33 countries, including Japan, China, Papua New Guinea and the Americas [5]. While prevalence is lower in these areas, as we have seen in Victoria over the last decade, case numbers can quickly increase, and the disease can spread to new areas, due to currently unknown mechanisms [4]. Developing a greater range of region-specific models could facilitate timely responses to future outbreaks.

Due to the similarity of the model structures, we were able to efficiently explore model differences, but the limitations of the models—particularly their infrequent use of data—restricted our ability to make comparisons regarding *M. ulcerans* dynamics. Nonetheless, our systematic review has identified gaps and we are able to make suggestions for future modelling work.

This review did not include systematic searches in languages other than English, which may have led to the omission of relevant studies, potentially from other contexts. Additionally, our focus on mechanistic transmission models may have constrained our findings; exploring other disease models, such as spatial or statistical frameworks, could yield further insights. It was also clear that our initial database choices did not encompass all the existing *M. ulcerans* modelling literature, although we attempted to overcome this with forwards and backwards searches.

This is the first systematic review of mechanistic transmission models of *M. ulcerans*. We aimed to investigate the transmission model structures and parametrisation methods that have been used to describe *M. ulcerans* transmission dynamics. We collated models, compared structures and analysis techniques, and concluded that while existing models provide a foundation, large gaps still exist in the field. We also sought to determine the relevance of existing model structures to observed transmission dynamics in Victoria, Australia. Few models have been developed specifically for the Victorian setting, and none of these model structures were validated with empirical data. While uncertainties in epidemiology in both the Central and West African and Victorian contexts complicate comparisons, differences in proposed dynamics between settings [7] suggest that models cannot be directly applied to different regions. Therefore, a large gap remains in mechanistic modelling of Buruli ulcer dynamics in Victoria, particularly with regard to human disease burden. Such models could be key in uncovering characteristics of the disease and identifying approaches to Buruli ulcer control.

## Supporting information

S1 Appendix Full search strategy. Full search strings for each of the four databases searched (PubMed, Scopus, Embase classic + Embase, CAB abstracts (CAB direct)).
(PDF)

S2 Appendix Quality review. Contains a table and discussion of the quality review for each included study, following a modified Philips Checklist.
(PDF)

S3 Appendix Full compartment model diagram. Contains Fig 3 from the main text with the addition of model ID labels showing which models used each compartment and transition/transmission routes.
(PDF)

S4 Appendix Applied analyses: fitted parameter values. Contains a table comparing the fitted values of like parameters from included studies that applied their models to epidemiological data.
(PDF)

S1 PRISMA Checklist PRISMA 2020 expanded checklist. Contains a table of recommended systematic review elements and text excerpts from this review where we have met those recommendations.
(PDF)
