# Supplementary material for: Transmission models of Mycobacterium ulcerans: A systematic review
Source: PLoS Negl Trop Dis. 2025 Aug 19;19(8):e0013376. doi: 10.1371/journal.pntd.0013376 (PMC12364374; doi:10.1371/journal.pntd.0013376)
Supplement: S1 Appendix — Full search strings for each of the four databases searched (PubMed, Scopus, Embase classic + Embase, CAB abstracts (CAB direct)). (PDF) [file pntd.0013376.s001.pdf]

S1 Appendix. Full search strategy.

PubMed

((buruli[Title/Abstract] OR mycobacterium[Title/Abstract] OR bairnsdale[Title/Abstract] OR mossman[Title/Abstract] OR searls') AND(ulcer*) AND (model*[Title/Abstract] OR paramet*[Title/Abstract] OR simulat*[Title/Abstract] OR program*[Title/Abstract] OR comput*[Title/Abstract])) OR ((buruli[MeSH Terms] OR mycobacterium[MeSH Terms] OR bairnsdale[MeSH Terms] OR mossman[MeSH Terms] OR searls'[MeSH Terms]) AND(ulcer*[MeSH Terms])AND (model*[MeSH Terms] OR paramet*[MeSH Terms] OR simulat*[MeSH Terms] OR program*[MeSH Terms] OR comput*[MeSH Terms]))

Scopus

TITLE-ABS-KEY (buruli OR mycobacterium OR bairnsdale OR mossman OR searls') AND (ulcer*)AND (model* OR paramet* OR simulat* OR program* OR comput*)

Embase classic + embase

((buruli or mycobacterium or bairnsdale or mossman or searls') and ulcer* and (model* or paramet* or simulat* or program* or comput*)).ti. or ((buruli or mycobacterium or bairnsdale or mossman or searls') and ulcer* and (model* or paramet* or simulat* or program* or comput*)).ab. or ((buruli or mycobacterium or bairnsdale or mossman or searls') and ulcer* and (model* or paramet* or simulat* or program* or comput*)).kw.

CAB abstracts (CAB direct)

ab:((buruli OR mycobacterium OR bairnsdale OR mossman OR "searls'") AND (ulcer*) AND (model* OR paramet* OR simulat* OR program* OR comput*)) OR Title:((buruli OR mycobacterium OR bairnsdale OR mossman OR "searls'") AND (ulcer*) AND (model* OR paramet* OR simulat* OR program* OR comput*)) OR Keyword:((buruli OR mycobacterium OR bairnsdale OR mossman OR "searls'") AND (ulcer*) AND (model* OR paramet* OR simulat* OR program* OR comput*))
