# Supplementary material for: Transmission models of Mycobacterium ulcerans: A systematic review
Source: PLoS Negl Trop Dis. 2025 Aug 19;19(8):e0013376. doi: 10.1371/journal.pntd.0013376 (PMC12364374; doi:10.1371/journal.pntd.0013376)
Supplement: S2 Appendix — Contains a table and discussion of the quality review for each included study, following a modified Philips Checklist. (PDF) [file pntd.0013376.s002.pdf]

S2 Appendix. Quality review.

Table 1: Modified Philips checklist for modelling study quality assessment. Y = yes, N = no, NA = not applicable. Questions on the original checklist have been left out where they were not applicable to any of the included studies.

Article ID	1	2	3	4	5	6	7	8	9	10	11	12	13	14	15	16	17	18	19	20
Is there a clear statement of the decision problem?	Y	Y	Y	Y	Y	Y	Y	Y	Y	Y	Y	Y	Y	Y	Y	Y	Y	Y	Y	Y
Is the objective of the model specified and consistent with the stated decision problem?	Y	Y	Y	Y	Y	Y	Y	Y	Y	Y	Y	Y	Y	Y	Y	Y	Y	Y	Y	Y
Is the primary decision maker specified? (who is going to use the model to make decisions)	N	N	N	N	N	N	N	N	N	N	N	N	N	N	N	N	N	N	Y	N
Is the perspective of the model stated clearly? (which costs and consequences are considered relevant)?	Y	Y	Y	Y	Y	Y	Y	Y	Y	Y	Y	Y	Y	Y	Y	Y	Y	Y	Y	Y
Are the model inputs consistent with the stated perspective? (and overall objective)	N	Y	N	Y	Y	N	Y	Y	Y	Y	Y	Y	Y	Y	Y	Y	Y	Y	Y	Y
Has the scope of the model been stated and justified? (the models limits and boundaries, statement of population, setting and time horizon the model applies to)	N	Y	N	Y	Y	Y	Y	Y	Y	N	Y	Y	Y	Y	Y	Y	N	Y	Y	Y
Are the outcomes of the model consistent with the perspective, scope and overall objective of the model? Capable of answering research question?	Y	Y	N	Y	Y	N	Y	Y	Y	Y	Y	Y	Y	Y	Y	Y	Y	N	Y	Y
Is the structure of the model consistent with a coherent theory of the health condition under evaluation?	Y	Y	Y	Y	Y	Y	Y	Y	Y	Y	Y	Y	Y	Y	Y	Y	Y	Y	Y	Y

Are the causal relationships described by the model structure justified appropriately?	Y	Y	N	Y	Y	Y	Y	Y	Y	Y	Y	Y	Y	Y	Y	Y	Y	Y	Y	Y
Are the structural assumptions transparent and justified?	Y	Y	Y	Y	Y	Y	Y	Y	Y	Y	Y	Y	Y	Y	Y	Y	Y	Y	Y	Y
Are the structural assumptions reasonable given the overall objective, perspective and scope of the model?	N	Y	Y	Y	Y	Y	Y	Y	Y	Y	Y	Y	Y	Y	Y	Y	Y	Y	Y	Y
Is the chosen model type appropriate given the decision problem and specified casual relationships within the model?	Y	Y	Y	Y	Y	Y	Y	Y	Y	Y	Y	Y	Y	Y	Y	Y	Y	Y	Y	Y
Is the time horizon of the model sufficient to reflect all important differences between the options?	Y	NA	Y	Y	Y	Y	Y	Y	Y	Y	Y	Y	Y	Y	Y	Y	N	Y	Y	Y
Are the time horizon of the model and the duration of treatment described and justified?	N	NA	N	N	N	Y	Y	N	Y	N	Y	N	N	N	N	N	N	N	Y	N
Do the disease states (state transition model) or the pathways (decision tree model) reflect the underlying biological process of the disease in question and the impact of interventions?	N	Y	N	N	N	N	N	N	N	N	N	N	Y	Y	N	N	N	Y	Y	Y
Are the data identification methods transparent and appropriate given the objectives of the model?	Y	Y	Y	N	Y	Y	Y	Y	Y	Y	Y	N	Y	Y	Y	Y	N	N	Y	N
Has particular attention been paid to identifying data for the important parameters of the model?	Y	Y	N	N	N	N	Y	N	Y	Y	Y	N	N	N	N	N	N	N	Y	N
Has the quality of the data been assessed appropriately?	N	N	N	N	N	N	N	N	N	N	N	N	N	N	N	N	N	N	N	N

Is the data modelling methodology based on justifiable statistical and epidemiological techniques?	NA	Y	NA	NA	NA	Y	NA	NA	Y	NA	Y	NA	NA	NA	NA	NA	NA	NA	Y	NA
Have all data incorporated into the model been described and referenced in sufficient detail?	N	Y	N	Y	N	Y	Y	Y	Y	N	Y	N	Y	Y	Y	Y	N	N	N	N
Is the process of data incorporation transparent?	Y	Y	Y	Y	Y	Y	Y	Y	Y	Y	N	Y	Y	Y	Y	Y	Y	Y	Y	Y
If data have been incorporated as distributions, has the choice of distributions for each parameter been described and justified?	NA	NA	NA	NA	NA	Y	Y	NA	NA	NA	NA	NA	NA	NA	NA	NA	NA	NA	NA	NA
Have methodological uncertainties been addressed by running alternative versions of the model with different methodological assumptions?	N	Y	N	N	N	N	Y	N	Y	N	Y	N	N	N	N	N	N	N	Y	Y
Has heterogeneity been dealt with by running the model separately for different subgroups?	N	N	N	N	N	N	Y	N	N	N	N	N	N	N	N	N	N	N	N	N
Are the methods of assessment of parameter uncertainty appropriate?	N	N	N	N	N	N	Y	N	Y	N	N	N	N	N	N	N	N	N	N	N
If data are incorporated as point estimates, are the ranges used for sensitivity analysis stated clearly and justified?	N	N	N	N	N	Y	Y	N	Y	N	Y	N	N	N	N	N	N	N	N	Y
Are any counter-intuitive results from the model explained and justified?	NA	Y	NA	NA	NA	NA	NA	NA	NA	NA	NA	NA	NA	NA	NA	NA	NA	NA	NA	NA
If the model has been calibrated against independent data, have any differences been explained and justified?	NA	NA	NA	NA	NA	N	NA	NA	NA	NA	NA	NA	NA	NA	NA	NA	NA	NA	NA	NA

Have the results been compared with those of previous models and any differences in results explained?	N	N	N	N	N	N	N	N	N	N	N	N	N	N	N	N	N	N	N	N

A quality assessment was conducted as part of this systematic review in the form of a modified Philips checklist. Characteristics aligning with points in the checklist have been discussed in the main text. The others were less critical for this review's purpose and are briefly discussed here. The included studies all provided clear statements of the problem, the study objectives and the perspectives and structural assumptions of the model. Methods of data identification and incorporation into the model were generally transparent. However, we found that the use of data could be improved in many cases. Some model parameter values were assumed rather than derived from real-world data. In instances where data was used to inform parameters, it often came from previous modelling papers rather than directly from epidemiological studies. Additionally, a few studies appeared to overfit the models, leading to values that seemed biologically implausible. As mentioned in the main text, most of the models also were not tested against real-world data, which reduced their validity and potential impact. All of the authors that conducted an applied analysis fit their model output incidence or prevalence directly to data on incidence/prevalence of *M. ulcerans*. However, such data can be prone to error and none of the included studies discussed the quality of the data used. All of these models fit quite well to their respective data sets, although only paper ID 2 made it clear how these data were collected (and therefore what potential biases might be).

As discussed in the main text, the biological processes behind Buruli ulcer remain unclear. Thus, the model objectives were typically theoretical, and so the primary decision makers or those who were going to use the model were never specified. For the same reason, it was also difficult to build a model to adequately reflect the biological pathways, although some studies more faithfully represented current knowledge than others.
