# Supplementary material for: Transmission models of Mycobacterium ulcerans: A systematic review
Source: PLoS Negl Trop Dis. 2025 Aug 19;19(8):e0013376. doi: 10.1371/journal.pntd.0013376 (PMC12364374; doi:10.1371/journal.pntd.0013376)
Supplement: S3 Appendix — Contains Fig 3 from the main text with the addition of model ID labels showing which models used each compartment and transition/transmission routes. (PDF) [file pntd.0013376.s003.pdf]

S3 Appendix. Full compartment model diagram

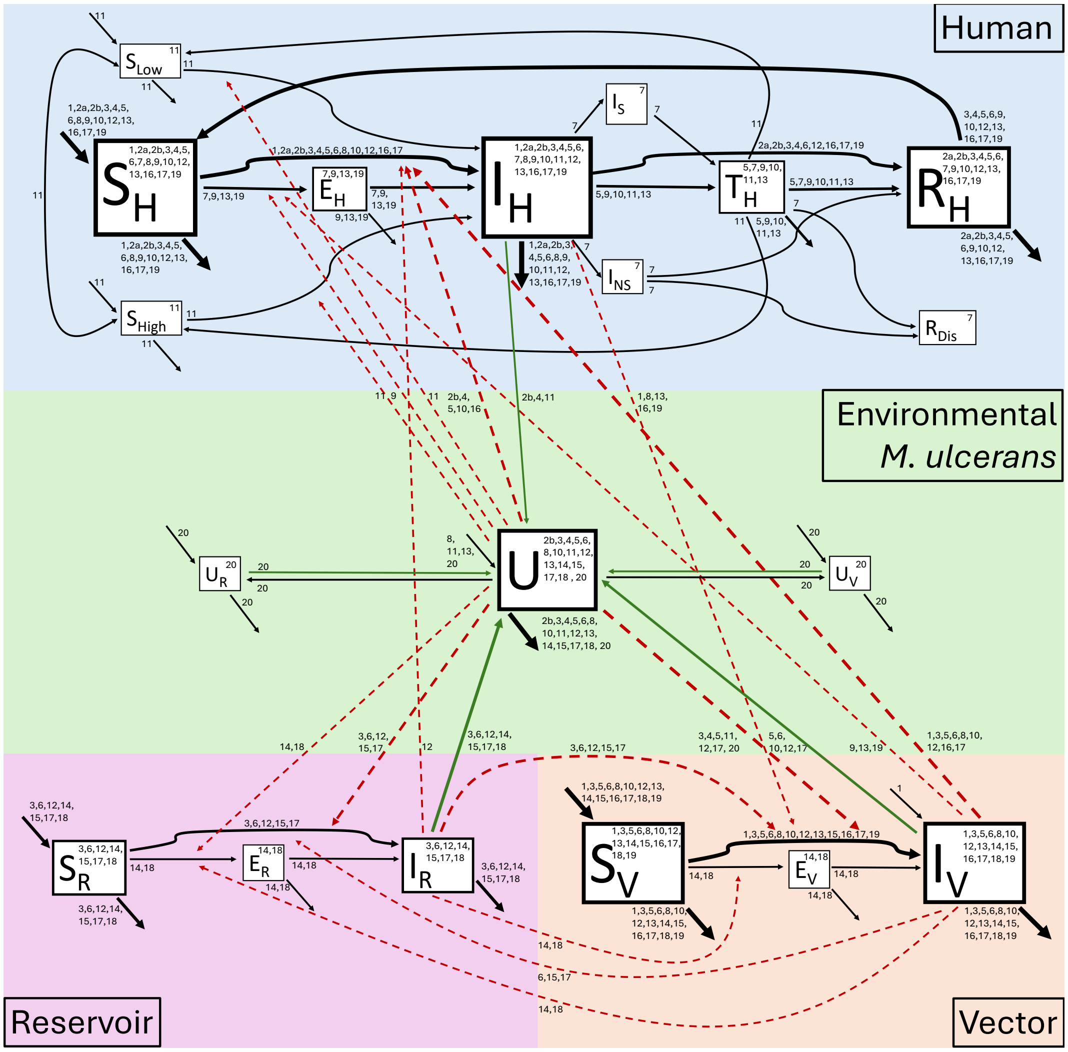

Figure 1: Compartment model diagram depicting the frequency of compartments in reviewed models and transition/transmission pathways. Compartments are susceptible (S), exposed (E), infectious (I), treated (T) and removed (R) with subscripts H for humans, R for reservoirs, V for vectors and compartment U for environmental *M. ulcerans*. S_{Low} and S_{High} represent susceptible individuals at low and high risk, respectively. I_S and I_{NS} represent infectious individuals seeking treatment or not seeking treatment. R_{Dis} represents individuals who acquire a disability as a result of having Buruli ulcer. U_R and U_V represent reservoir and vector associated bacteria. Compartment box sizes represent the relative frequency of these compartments in the reviewed models. Black arrows indicate transitions of individuals between compartments, red dashed arrows represent *M. ulcerans* transmission routes and green arrows represent shedding of *M. ulcerans* from infectious compartments into the environment. Thickness of the arrows represent the relative frequency of pathways in the reviewed models. The IDs for the models containing each pathway and compartment are shown next to arrows and in boxes.
