# Supplementary material for: Transmission models of Mycobacterium ulcerans: A systematic review
Source: PLoS Negl Trop Dis. 2025 Aug 19;19(8):e0013376. doi: 10.1371/journal.pntd.0013376 (PMC12364374; doi:10.1371/journal.pntd.0013376)
Supplement: S4 Appendix — Contains a table comparing the fitted values of like parameters from included studies that applied their models to epidemiological data. (PDF) [file pntd.0013376.s004.pdf]

S4 Appendix. Applied analyses: fitted parameter values

Table 1: Applied analyses: fitted parameter values. These values have been converted (where the data were made available) so that units correspond between papers. The *M. ulcerans* (MU) transmission rates to humans have been reported in the same form as in the original articles. Transmission rate (force of infection for ID 9) form has been reported for each article.

Parameter	Ghana		Cameroon	
	Paper ID 6	Paper ID 11	Paper ID 9	Paper ID 19
Average human lifespan (years)	60.9	61	30	55.5
Human birth rate (per capita per day)	4.5×10^{-5}	$\approx 1.0 \times 10^{-4}$	9.4×10^{-5}	$\approx 8.9 \times 10^{-5}$
MU transmission rates to humans	Frequency dependent 0.1	Michaelis–Menten function 1.00×10^{-10} (low risk), 1.47×10^{-5} (high risk)	Density dependent (force of infection) 1.27×10^{-4} (environmental), 1.86×10^{-6} (vector)	Frequency dependent 2.2×10^{-3}
Human latency period (months)	NA	NA	3–5	24
Human duration of infectiousness (months)	0.6	3.5	2–6	16
Environmental MU decay rate (per day)	0.4	5.63×10^{-3}	NA	NA
